# Impact of shear stress on sacral pressure injury from table rotation during laparoscopic colorectal surgery performed in the lithotomy position

**DOI:** 10.1038/s41598-024-60424-9

**Published:** 2024-04-28

**Authors:** Kyota Tatsuta, Mayu Sakata, Kosuke Sugiyama, Tadahiro Kojima, Toshiya Akai, Katsunori Suzuki, Kakeru Torii, Yoshifumi Morita, Hirotoshi Kikuchi, Yoshihiro Hiramatsu, Kiyotaka Kurachi, Hiroya Takeuchi

**Affiliations:** 1 Department of Surgery, Hamamatsu University School of Medicine, 1-20-1, Handayama, Higashi-ku, Hamamatsu, Shizuoka 431-3192 Japan; 2 Division of Surgical Care, Morimachi, Hamamatsu University School of Medicine, 1-20-1, Handayama, Higashi-ku, Hamamatsu, Shizuoka 431-3192 Japan; 3 Department of Perioperative Functioning Care and Support, Hamamatsu University School of Medicine, 1-20-1, Handayama, Higashi-ku, Hamamatsu, Shizuoka 431-3192 Japan

**Keywords:** Gastroenterology, Gastrointestinal diseases

## Abstract

This study aimed to evaluate the impact of shear stress on surgery-related sacral pressure injury (PI) after laparoscopic colorectal surgery performed in the lithotomy position. We included 37 patients who underwent this procedure between November 2021 and October 2022. The primary outcome was average horizontal shear stress caused by the rotation of the operating table during the operation, and the secondary outcome was interface pressure over time. Sensors were used to measure shear stress and interface pressure in the sacral region. Patients were divided into two groups according to the presence or absence of PI. PI had an incidence of 32.4%, and the primary outcome, average horizontal shear stress, was significantly higher in the PI group than in the no-PI group. The interface pressure increased over time in both groups. At 120 min, the interface pressure was two times higher in the PI group than in the no-PI group (PI group, 221.5 mmHg; no-PI group, 86.0 mmHg; *p* < 0.01). This study suggested that shear stress resulting from rotation of the operating table in the sacral region by laparoscopic colorectal surgery performed in the lithotomy position is the cause of PI. These results should contribute to the prevention of PI.

## Introduction

Pressure injury (PI) causes occlusion of blood flow and can affect the skin, soft tissue, muscle, and bone. It leads to the development of localized ischemia, tissue inflammation, tissue anoxia, and necrosis^1^. Surgery is a risk factor for PI^2^. Surgery-related PI is reportedly caused by pressure, shear stress, or friction tissue forces, which can occur because of prolonged periods of immobility during an operation^2,3^. Surgery-related PI leads to longer hospital stays and higher hospital costs^4,5^.

The rate of surgery-related PI differs according to the surgical position. The lithotomy position is recognized as a high-risk position for surgery-related PI^6,7^. In recent years, laparoscopic and robot-assisted colorectal surgeries have become common^8–10^. Laparoscopic or robot-assisted surgery performed in the lithotomy position requires the utilization of positioning devices and rotation of the operating table. Rotation of the operating table can cause shear stress^11^. Therefore, the risk of surgery-related PI is expected to increase further when rotation of the operating table is added to the lithotomy position. Several reports have shown surgery-related PI caused by shear stress due to the rotation of the operating table^12,13^. However, no studies have specifically investigated the effect of shear stress due to the rotation of the operating table on surgery-related PI.

We hypothesized that shear stress associated with the rotation of the operating table is strongly related to the cause of surgery-related PI in laparoscopic colorectal surgery performed in the lithotomy position. Several areas of the body are considerably affected by PI. The most common postoperative sites where PI is reported to occur are the occipital skull, scapula, elbows, sacral region, and heels. Surgery-related PI in the sacral region is more likely to be fatal^14^. This study aimed to evaluate the impact of shear stress on surgery-related PI in the sacral region by laparoscopic colorectal surgery performed in the lithotomy position.

## Methods

### Study design and patient population

This prospective cohort study recruited and enrolled all patients who underwent laparoscopic colorectal surgery in lithotomy position between November 2021 and October 2022. Among these, we excluded loop colostomy, which does not require rotation of the operating table, and total proctocolectomy, which requires various directions or angles of rotation of the operating table in a single surgery. Robotic-assisted surgery was excluded as it was in the introductory phase. The study design was approved by the Institutional Review Board of Hamamatsu University School of Medicine (IRB number: 20-226) and registered in UMIN-CTR Clinical Trial Registry (UMIN000051051). All methods were performed in accordance with the relevant guidelines and regulations. Written informed consent was obtained from all patients whose physical characteristics were assessed.

### Procedures

**Figure 1:**
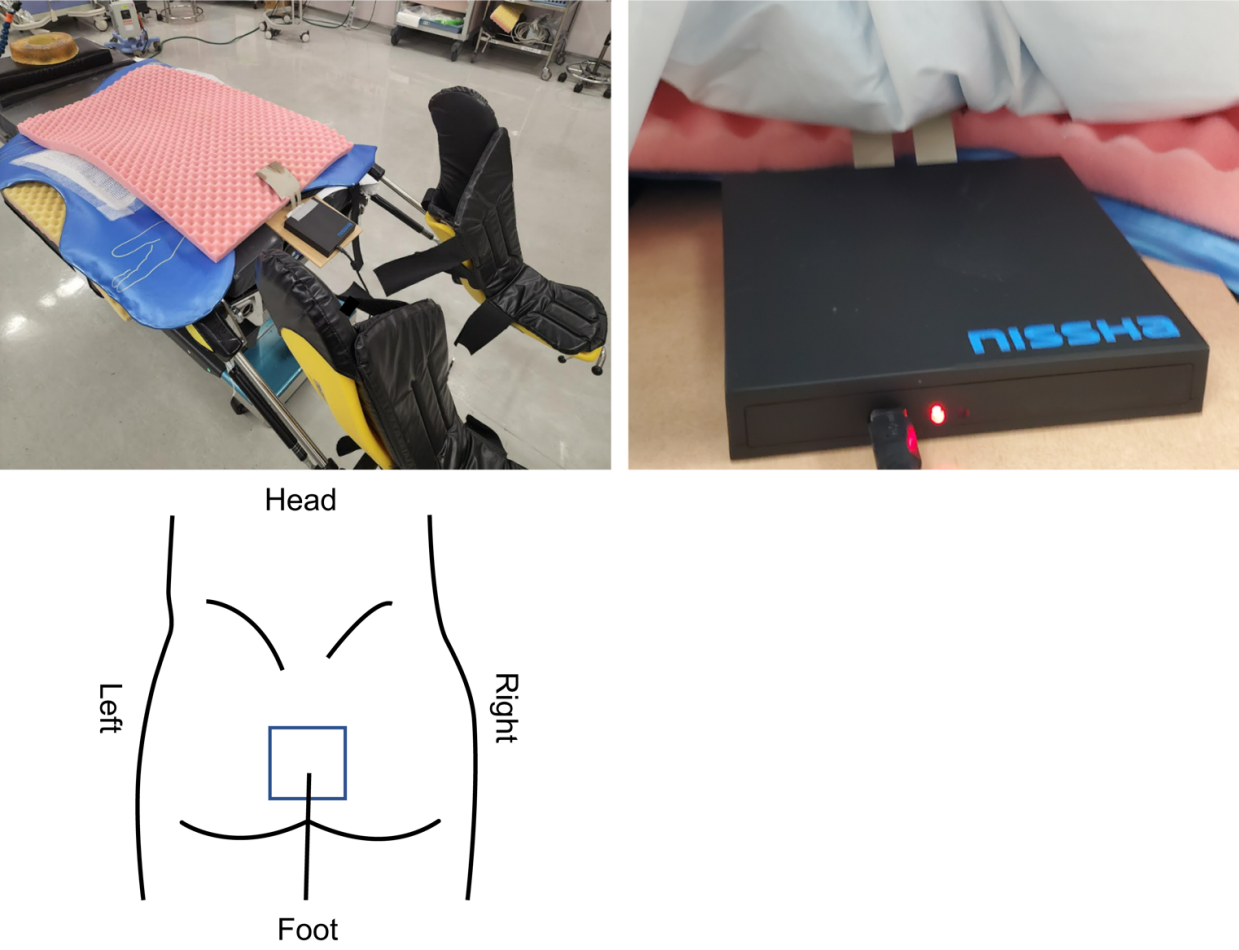
The method of setting up the pressure and shear stress sensor. After the lithotomy position, the sensor is set in alignment with the patient’s sacral region.

Pressure and shear force sensors (Nissha Co., Ltd., Kyoto, Japan) were used to measure the horizontal and interfacial pressures in the sacral region. This sensor can measure an area of 44 mm × 66 mm (11 × 11 cells) every 0.01 s. Changes in the pressure values were recorded consecutively and saved as numeric data. After the lithotomy position, the sensor was placed on top of the positioning devices and pressure redistribution urethane foam (Fig. 1). The surgical team measured and recorded the horizontal and interface pressure distributions in the sacral region in both the flat and tilted positions during the operation. The tilt was 15° in the lower-head position and 15° in the lower-right position, and the position was continued for 120 min. After returning to the flat position for 5 min, the operating table was repositioned. This series of the rotation of the operating table was repeated until the surgical procedure was completed. This protocol was determined in accordance with our previous study to prevent well-leg compartment syndrome^15^. The recording time was defined as the time from the start of surgery to bowel resection to minimize getting the sensor soiled by the surgical procedure.

### Definition of PI in the sacral region

PI was evaluated in the operating room immediately after surgery by multiple members of the surgical team for redness in the sacral region. Patients with skin redness were defined as the PI group. The PI group included patients with pressure ulcers (non-blanchable redness) and reactive hyperemia (blanchable redness). Pressure ulcer was classified based on the National Pressure Ulcer Advisory Panel (NPUAP)^16^. In cases of pressure ulcers, treatment was continued based on the international clinical practice guidelines for the prevention and treatment of pressure ulcers and injuries^17^.

### Analysis of horizontal pressure, interface pressure, and shear stress

The force data of the tri-axes are defined as “X-axis” for the lateral pressure within horizontal direction, “Y-axis” for the longitudinal pressure within horizontal direction, and “Z-axis” for the interface pressure. The vector component was calculated from the numerical data of the X, Y, and Z axes of each cell. The direction of the horizontal pressure was calculated as the fundamental unit vector and represented the direction of the arrow. On the X-axis, positive corresponds to the left side and negative to the right side; on the Y-axis, positive corresponds to the foot side and negative to the head side. The horizontal shear stress was calculated as the difference between adjacent cells. These analyses were performed for each cell, four areas (4 × 4 cells), and the entire area.

### Outcome measurements

The primary outcome was the average horizontal shear stress in the head and right-down tilt position during the operation. Secondary outcomes were the direction for horizontal pressure in the sacral region, the change over time in horizontal shear stress and the interface pressure in the sacral region. The change over time was evaluated in the flat position, and the head and right-down tilt positions were evaluated every 30 min up to 120 min. Additional secondary outcomes included pre-operative patient characteristics and intra-operative outcomes. In the preoperative patient’s characteristics, the areas of abdominal visceral fat, subcutaneous fat, and psoas major muscle were calculated from a computed tomography (CT) image acquired at the level of L3 using SYNAPSE VINCENT (Fujifilm, Japan). Skin and subcutaneous tissue thicknesses were measured at the thinnest part of the sacral region. The prognostic nutritional index (PNI) was calculated as 10 × serum albumin (g/dL) + 0.005 × total lymphocyte counts (per mm^3^)^18^. As a post-hoc analysis, we evaluated the distribution of shear stress over the entire area.

### Statistical analyses

Statistical analyses were performed using JMP® 16 software (SAS Institute Inc., Cary, NC, USA). The distribution features are presented as mean ± standard error (SE) or median and interquartile range (IQR) for variables with skewed distribution or frequency (proportion [%]). The medians and ranges were calculated, and differences were identified using the Mann–Whitney U test. Categorical data were expressed as frequencies and proportions and analyzed using Fisher’s exact test. Cosine similarity was used to compare the horizontal pressure direction. The cosine similarity is normalized to a range of -1 to 1, where 1 indicates that the horizontal pressure directions are perfectly similar, and -1 indicates that they are not perfectly similar. Statistical significance was set at *P* < 0.05.

## Results

### Patients

During the enrollment period, 37 of the 38 patients were included and divided into 2 groups, with or without the presence of PI. One patient was excluded owing to sensor failure. The characteristics and intraoperative outcomes of the study participants are summarized in Table 1. The incidence of PI was 32.4% (pressure ulcer, 1; reactive hyperemia, 11). No differences were observed in clinical characteristics and intraoperative outcomes. In 83.8% of cases (PI group: 91.7%, no-PI group: 80.0%), surgery was completed within 120 min from the start of the tilt position.

**Table 1:** Clinical characteristics and intraoperative outcomes.

	PI n = 12	no PI n = 25	*P*-value
Age, years, median (range)	65.0 (39–74)	67.0 (47–81)	0.475
Sex, n (%)			1.000
Male	7 (58.3)	15 (60.0)	
Female	5 (41.7)	10 (40.0)	
Body mass index, kg/m^2^, median (range)	26.1 (15.8–31.6)	22.9 (18.4–37.1)	0.211
ASA, ≥ 3 (%)	1 (8.3)	2 (8.0)	1.000
Current smoker, n (%)	1 (8.3)	3 (12.0)	1.000
Past history, n (%)			
Diabetes mellitus	3 (25.0)	6 (24.0)	1.000
Continuous corticosteroids treatment	0 (0)	1 (4.0)	1.000
Indication for resection, n (%)			0.550
Malignancy	11 (91.7)	24 (96.0)	
Benign	1 (8.3)	1 (4.0)	
Preoperative PNI, median (range)	47.6 (40.5–55.4)	46.8 (33.2–58.9)	0.783
Visceral fat at the level of L3, cm^2^, median (range)	137.8 (27.5–349.2)	133.7 (19.7–510.8)	0.808
Subcutaneous fat at the level of L3, cm^2^, median (range)	111.7 (4.0–290.8)	99.4 (11.6–268.7)	0.685
Psoas major muscle at the level of L3, cm^2^, median (range)	16.5 (8.0–28.4)	13.3 (6.7–25.4)	0.200
The skin and subcutaneous tissue thickness in the sacral region, mm, median (range)	14.5 (8.2–31.9)	12.1 (4.9–29.3)	0.119
Operation time, min, median (range)	286 (171–508)	252 (158–510)	0.506
Duration time in the lithotomy position, min, median (range)	333 (200–539)	295 (193–567)	0.559
Blood loss, ml, median (range)	25 (5–74)	20 (5–160)	0.987
Red blood cell transfusion, n (%)	0 (0)	1 (4.0)	1.000

*ASA* American Society of Anesthesiologists; *PNI* Prognostic nutritional index.

*P*-value < 0.05.

### Characteristics of PI

**Figure 2:**
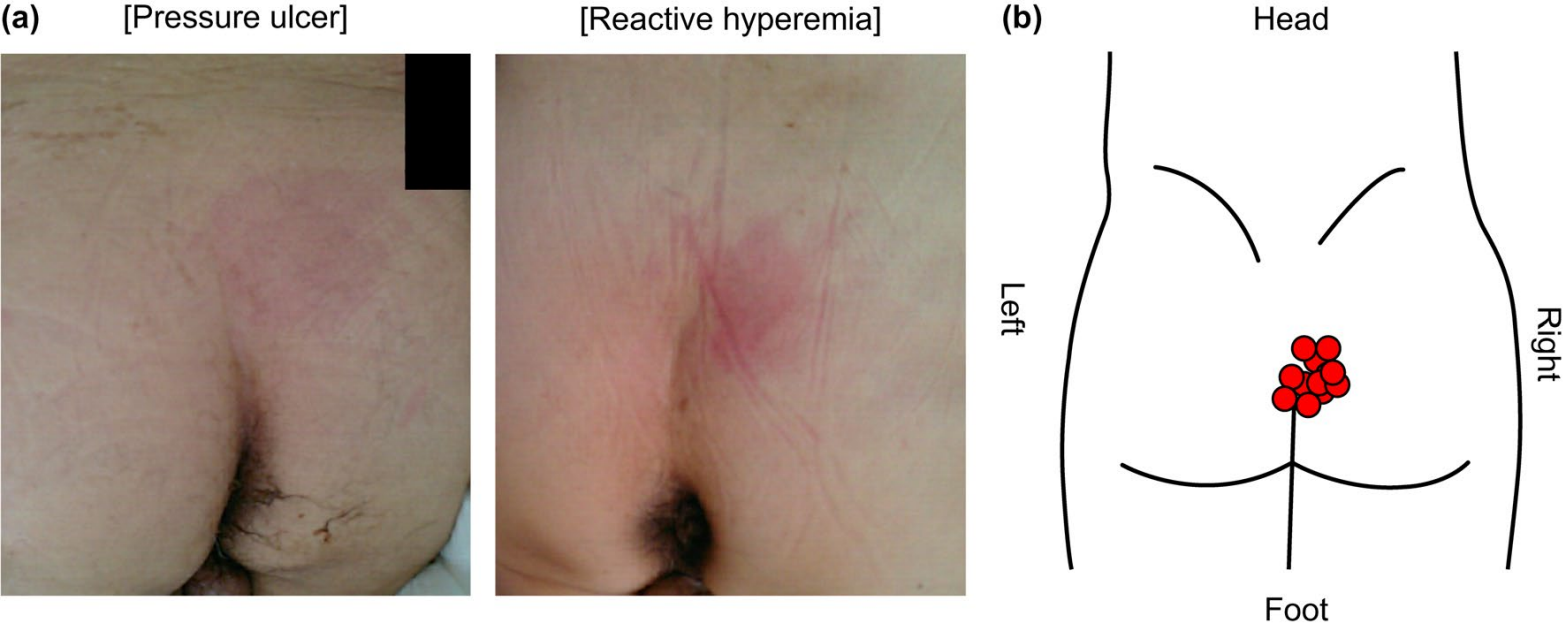
The site of occurrence of surgery-related pressure injury. (**a**) The representative pressure ulcer and reactive hyperemia cases are shown. (**b**) Each case mapped the center of the site of occurrence of surgery-related pressure injury.

PI mainly occurred on the right sacral region (Fig. 2). All patients with reactive hyperemia showed improvement in redness the following day. One case of pressure ulcer was treated with white petrolatum and healed within 3 days.

### Primary endpoint

**Figure 3:**
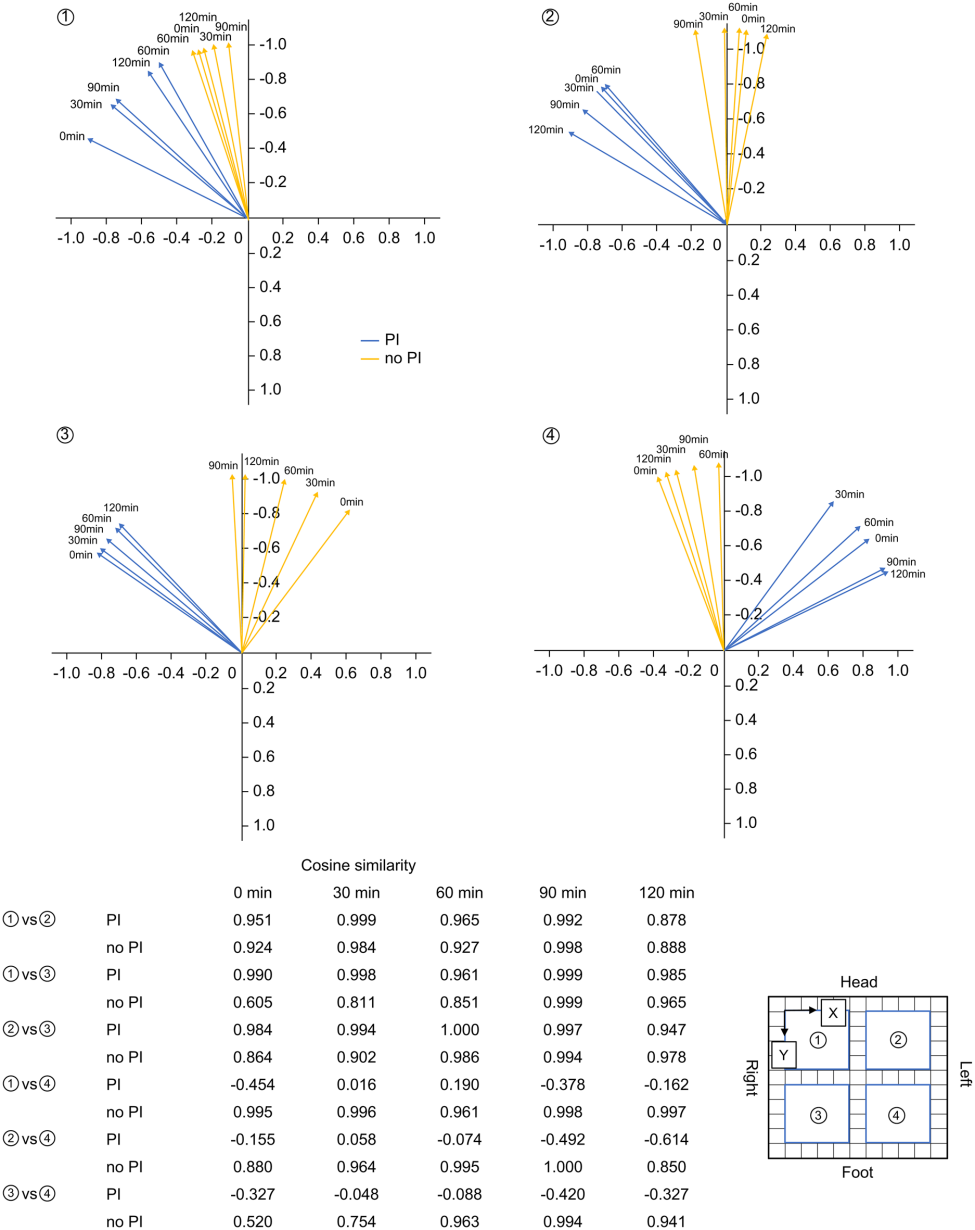
Horizontal shear stress in the sacral region. Heatmap of shear stress over the entire sacral region, rated from 0 to 100 mmHg. The change over time up to 120 min from the start of the tilt position was evaluated. PI, pressure injury.

Table 2 lists the results for the horizontal shear stress. The average horizontal shear stress in the head and right-down tilt position during the operation was significantly higher in the PI group than in the no-PI group on both the X and Y axes. A post-hoc analysis in which the distribution of shear stress over the entire area showed the PI group was higher shear stress on the right side of the sacral region (Fig. 3).

**Table 2:** Horizontal shear stress.

	PI n = 12	no PI n = 25	*P*-value
X axis shear stress			
Average in the tilt position during the operation, mmHg, median (range)	26.1 (5.9–67.4)	22.3 (5.9–54.2)	< 0.001
0 min, mmHg, median (range)	27.6 (5.8–49.8)	25.8 (10.3–44.0)	0.150
30 min, mmHg, median (range)	26.1 (8.8–61.7)	24.1 (8.8–39.6)	0.220
60 min, mmHg, median (range)	25.4 (8.8–52.8)	23.4 (5.9–54.2)	0.113
90 min, mmHg, median (range)	25.5 (7.3–54.2)	20.7 (8.8–38.1)	< 0.001
120 min, mmHg, median (range)	25.7 (5.9–67.4)	17.7 (5.9–46.9)	< 0.001
Y axis shear stress			
Average in the tilt position during the operation, mmHg, median (range)	33.3 (6.1–81.8)	25.5 (8.1–67.6)	< 0.001
0 min, mmHg, median (range)	38.1 (13.5–78.5)	29.0 (13.5–55.5)	< 0.001
30 min, mmHg, median (range)	34.7 (10.8–81.2)	24.6 (8.1–37.9)	< 0.001
60 min, mmHg, median (range)	33.3 (13.5–67.6)	27.6 (13.5–67.6)	< 0.001
90 min, mmHg, median (range)	30.7 (6.1–73.7)	21.7 (8.1–43.3)	< 0.001
120 min, mmHg, median (range)	29.9 (8.8–66.3)	24.7 (8.1–54.1)	0.003

*P*-value < 0.05.

### Secondary endpoint

#### Direction for horizontal pressure for in the sacral region

**Figure 4:**
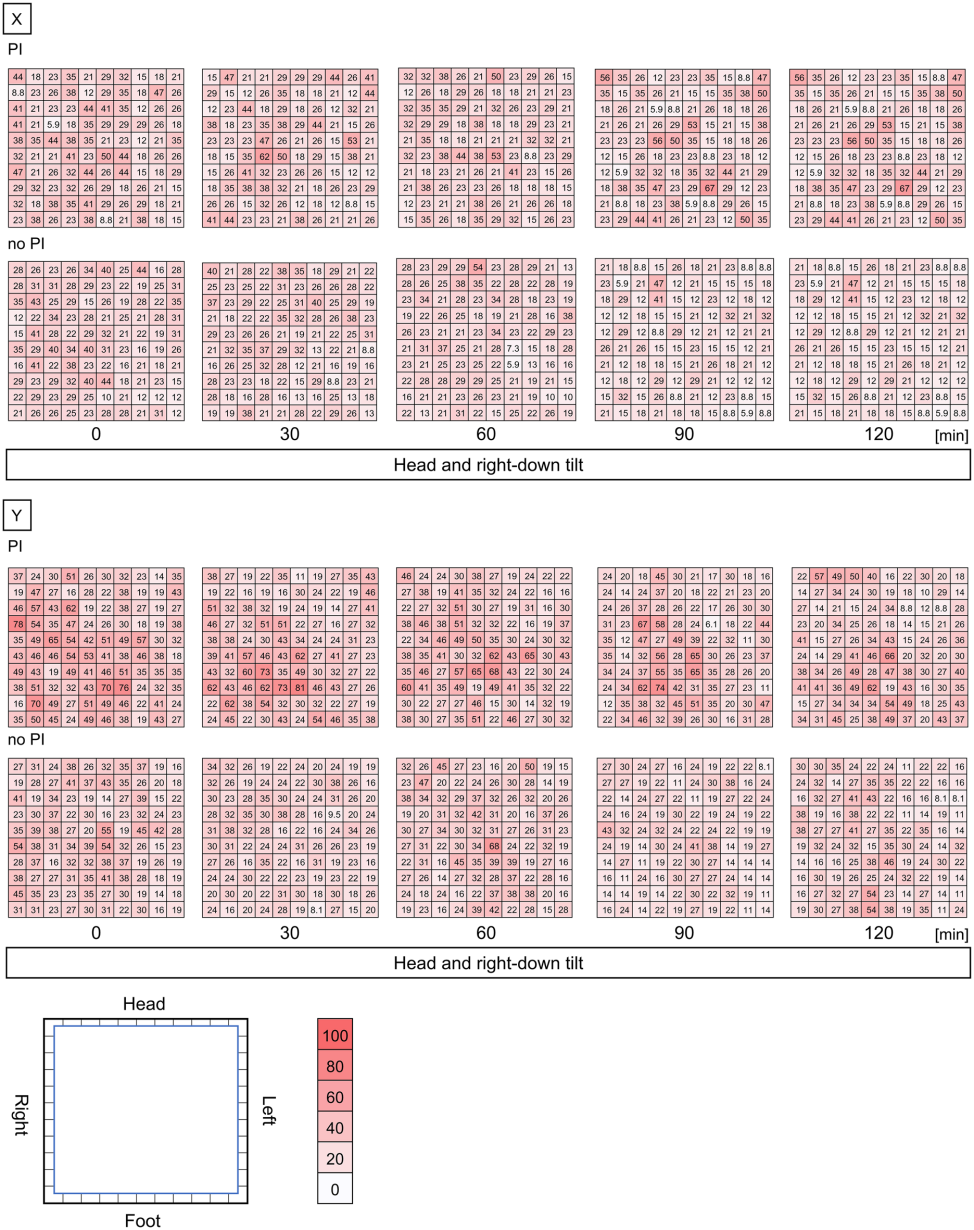
Direction for horizontal pressure for in the sacral region. The direction of horizontal pressure was analyzed in four separate areas (4 × 4 cells). The change over time up to 120 min from the start of the tilt position was evaluated. The similarity of vector components for each region was evaluated using cosine similarity over time. PI, pressure injury.

Figure 4 shows the direction of the horizontal pressure over time. In the no-PI group, the component of the longitudinal pressure in the horizontal direction was strong in each region, and most of the horizontal pressure was directed toward the head side. In contrast, in the PI group, the horizontal pressure was directed toward the right temporal direction in areas 1, 2, and 3, but toward the left temporal direction only in area 4. For the no-PI group, a correlation was noted in the horizontal pressure direction in all areas at all times. However, the PI group had correlations between areas 1 and 3, whereas area 4 had no correlation with the other areas in the horizontal direction.

#### The change of horizontal shear stress over time

Over time, the shear stress on the Y-axis was significantly higher in the PI group at all times. The shear stress on the X-axis was statistically different from 90 min onward (Table 2).

#### Interface pressure in the sacral region

**Figure 5:**
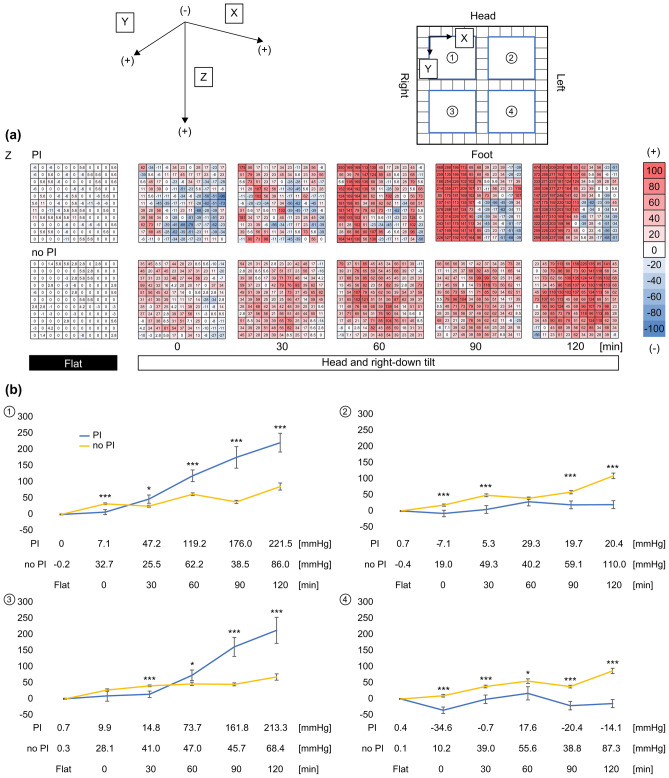
Interface pressure in the sacral region. (**a**) Heatmap of interface pressure over the entire sacral region, rated from -100 to 100 mmHg. (**b**) Interface pressure was analyzed in four separate areas (4 × 4 cells). Median ± standard error values were represented. *p < 0.05, **p < 0.005, *** *p* < 0.0005. PI, pressure injury.

Figure 5a shows a heat map of the interface pressure changes over time in the sacral region. The interface pressure increased over time in both groups. In the no-PI group, the interface pressure increased uniformly in all areas. However, in the PI group, the interface pressure increased dramatically in areas 1 and 3, whereas no increase in pressure was observed in areas 2 and 4. Figure 5b shows the numerical data of the changes in the interface pressure over time in each area. In areas 1 and 3, the PI group showed significantly higher interface pressure than the no-PI group 60 min after the start of lithotomy position, followed by a more dramatic increase. At 120 min after the start of the lithotomy position, the interface pressure was twice as high in the PI group as in the no-PI group (PI group, area 1: 221.5 mmHg; no-PI group, area 1: 86.0 mmHg; *p* < 0.01).

## Discussion

This was a prospective observational study investigating the impact of shear stress on surgery-related PI in laparoscopic colorectal surgery performed in the lithotomy position. The shear stress was significantly higher in the PI group and tended to be higher on the right side of the sacral region. Moreover, the PI group showed twice as much interface pressure in the sacral region as the no-PI group. This is the first study to demonstrate the impact of shear stress in the sacral region in the lithotomy position on the occurrence of surgery-related PI. This study significantly contributes to the prevention of surgery-related PI.

The effect of shear stress on the development of pressure ulcers has been widely reported^19^. However, previous studies on the relationship between shear stress and PI have been limited to the quantitative measurement of pressure and shear stress on the body of wheelchair users^20,21^ or on foot ulcers in patients with diabetes mellitus^22–24^. No reports have evaluated the relationship between surgery-related PI and shear stress. As we hypothesized, shear stress due to the rotation of the operating table, the primary endpoint of this study, was shown to significantly impact surgery-related PI. According to past reports, a shear stress of 3.1 kPa (approximately 23.3 mmHg) applied to the sacral region affects blood flow reduction in the sacral region^21,25^. In the present study, the average X and Y axes values were as high as 26.1 mmHg and 33.3 mmHg, respectively. The bias in the direction of the horizontal pressure in each area is considered the cause of the shear stress development. This bias started at the beginning of the tilt position and continued over time. The shear stress, especially on the Y-axis, was significantly greater in the PI group over time from the beginning of the tilt position. In addition, both the sites of high shear stress and occurrence of surgery-related PI were on the right side of the sacrum. Further, this result indicates that shear stress affects surgery-related PI.

Regarding the interface pressure, the results were also strongly influenced by the rotation of the operating table. The results of a previous study on interface pressure in the lithotomy position without rotation showed that the interface pressure was 93.3 mmHg in the sacral region over time^7^. In our study, the interface pressure in the sacral region of the no-PI group ranged from 68.4 to 110 mmHg. Surprisingly, the PI group showed a left–right difference in interface pressure in the sacral region, with the right side of the sacral region being > 200 mmHg. Previous studies have shown that the primary cause of pressure ulcers is ischemia produced by external pressures greater than capillary pressure (12–32 mmHg), and a constant pressure of 70 mmHg applied for 2 h produced ischemic changes^26–28^. In the PI group, an interface pressure > 200 mmHg on the right side of the sacral region was extremely abnormal. Similar to the mechanism of shear stress development, the bias in the direction of the horizontal pressure by each area is considered to be the cause of interface pressure development. We believe that it is crucial to elucidate the reason for the bias in the direction of the horizontal pressure.

Considering that the same positioning devices are used in all surgeries, we assume that this bias may reflect differences in the orientation and tilt of the patient’s body axis that occur when using positioning devices or that are caused by the patient’s body balance. Generally, the nutritional status, a history of diabetes mellitus, a high body mass index, prolonged surgery, and massive blood loss are considered risk factors for surgery-related PI^2,29,30^. The present study examined results of these previous studies using an index reflecting nutritional and body mass indexes in detail. We used PNI for nutritional indices^31^ and L3 levels of visceral fat, subcutaneous fat, and the psoas major muscles for high body mass index^32^. However, no difference was found in the clinical characteristics and intraoperative outcomes. There may be reasons for the occurrence of surgery-related PI specific to laparoscopic or robot-assisted surgery in the lithotomy position.

The incidence of surgery-related PI in this study was found to be 32.4%, which was higher than the incidence of PI in the lithotripsy position reported to be 25.9% in a previous study^33^. The incidence of PI in this study was probably higher because this study was limited to laparoscopic colorectal surgery with rotation of the operating table. On the other hand, the frequency of pressure ulcer was 2.7%, which was lower than that reported previously^34^. However, the frequency of surgery-related PI in the lithotomy position is much higher than the general incidence of surgery-related PI (6.3%)^3^. Thus, various preventive measures should be taken to reduce surgery-related PI in the lithotomy position.

Although this study showed that shear stress is associated with surgery-related PI, we were not able to make any recommendations regarding preventive measures for such injuries. A recent systematic literature review indicates that PI risk assessment and pressure redistribution using dressings are recommended^35^. In addition, recent clinical trials have examined the type of dressing material and showed that multi-layered silicone foam is more efficacious than transparent polyurethane film in preventing PI caused by surgical positioning^36^. Further research specific to the lithotomy position is desirable based on these preventive measures.

Our study had several limitations. First, it was a single-center study with a small sample size. Our participants represented a specific patient population, thus limiting the generalizability of our findings. Future studies with larger sample sizes should be conducted to confirm the results of this study and explore the effects of shear stress and interface pressure over longer periods. Second, the analysis in this study was limited because it was not performed for the entire lithotomy position. However, since only one patient (8.3%) in the PI group returned to the flat position and performed a second tilt position, we did not believe that this would significantly affect the results of this study. Third, we examined cases of laparoscopic surgeries performed in the lithotomy position that required rotation of the operating table. Therefore, the results of this study cannot be applied to cases that do not require rotation or that are rotated in different directions or angles. Fourth, this study did not consider other factors associated with pressure ulcers, such as perfusion, oxygenation, skin moisture, and body temperature. The influence of tissue damage differs by the tissue type and may be influenced by microclimate, perfusion, systemic comorbidities, and localized conditions of soft tissues, which are affected by sustained mechanical loading^37^. Therefore, a prospective study that includes various factors involved in PI development is required to validate the present study's results.

## Conclusion

This study provides evidence that shear stress in the sacral region due to rotation of the operating table in laparoscopic colorectal surgery performed in the lithotomy position is the cause of surgery-related PI. These results emphasize a contribution towards the prevention of surgery-related PI.

## Data Availability

The datasets generated during and/or analyzed during the current study are available from the corresponding author on reasonable request.
